# NMR Derived Model of GTPase Effector Domain (GED) Self Association: Relevance to Dynamin Assembly

**DOI:** 10.1371/journal.pone.0030109

**Published:** 2012-01-12

**Authors:** Swagata Chakraborty, Supriya Pratihar, Ramakrishna V. Hosur

**Affiliations:** Department of Chemical Sciences, Tata Institute of Fundamental Research, Mumbai, India; Consejo Superior de Investigaciones Cientificas, Spain

## Abstract

Self-association of dynamin to form spiral structures around lipidic vesicles during endocytosis is largely mediated by its ‘coiled coil’ GTPase Effector Domain (GED), which, in vitro, self-associates into huge helical assemblies. Residue-level structural characterizations of these assemblies and understanding the process of association have remained a challenge. It is also impossible to get folded monomers in the solution phase. In this context, we have developed here a strategy to probe the self-association of GED by first dissociating the assembly using Dimethyl Sulfoxide (DMSO) and then systematically monitoring the refolding into helix and concomitant re-association using NMR spectroscopy, as DMSO concentration is progressively reduced. The short segment, Arg109 - Met116, acts as the nucleation site for helix formation and self-association. Hydrophobic and complementary charge interactions on the surfaces drive self-association, as the helices elongate in both the directions resulting in an antiparallel stack. A small N-terminal segment remains floppy in the assembly. Following these and other published results on inter-domain interactions, we have proposed a plausible mode of dynamin self assembly.

## Introduction

Self-association processes in nature are interesting aspects and worth detailed studies owing to their relevance in the design and understanding of functional units on a supramolecular level. Self-association processes can perturb biological functions, such as the formation of cataracts in the lens of the eye or amyloid fibrils associated with neurological diseases like Alzheimer's [1], [2]. Sometimes, self-association is a necessary step of biological function; self-interactions govern the assembly of oligomeric proteins [3] and various cellular functions are mediated through protein-protein association. A thorough understanding of how these proteins interact with themselves in solution to form self–aggregates is essential for fundamental understanding of the mechanism of their functions.

Our protein of interest, GTPase Effector Domain (GED) is the functional subunit of dynamin which is involved in receptor-mediated endocytic machinery in cells [4], [5]. Dynamin assembles to form a long rope which wraps around the necks of lipid vesicles and mediates their scission from parent membranes. Oligomerization of dynamin is known to be lipid-dependent [6] and the GTPase, middle and GTPase effector domains are involved in the self-assembly of dynamin [7], [8], [9]. The coiled coil assembly domain, GED [10] is essential for stimulating the GTP hydrolysis of dynamin, thereby acting as an internal GTPase Activating Protein (GAP). GED, intrinsically is known to form huge assemblies in vitro [11]. These oligomers are soluble in water and exist in equilibrium with the monomers, the equilibrium being largely favored towards the oligomer. Even at µM concentrations, the protein was found to exhibit the monomer – oligomer equilibrium, with the major population arising from the oligomeric species [11]. The monomers and oligomers, both are largely helical in nature. The TEM image of the GED oligomers shows wide diversity of shape and size [12], one of them being the elongated cylindrical shape. This sort of elongated shape of the GED oligomers invokes utmost interest as dynamin oligomers are known to form rope like structures around the neck of the vesicles. Hence, a proper understanding of the assembly process of GED in a lipid-water mixture like environment is of utmost importance in order to gain insights into the association process of dynamin. However, due to the large size of the GED oligomers (>5 MDa), structural characterization of the protein in the native state has remained as a major challenge. The large molecular weight of the oligomers provides a major hindrance in accessing any residue-level information on the topology or the association mechanism of GED. Thus, in order to probe the association process of GED, we have devised here an indirect strategy, wherein we monitor the stepwise association of the protein, starting from the denatured state created by polar aprotic organic solvent Dimethyl Sulfoxide (DMSO). We have monitored the structural and motional characteristics of the different equilibrium states created by 100%, 90%, 85%, 80%, 70% and 50% DMSO (vol/vol) which are supposed to provide molecular snapshots of the different intermediates in the association pathway of the protein.

DMSO is amphiphilic due to the presence of polar hydrophilic sulphoxide group and the nonpolar hydrophobic methyl group, and hence, at lower concentrations, it can be taken to imitate lipid-water mixture to certain extent in terms of polarity. Though homogeneous DMSO–water mixtures may not be exact replacements for lipid-water environment which have interfaces, we choose DMSO in place of real lipids since it also dissociates the assembly, thereby enabling step-wise NMR characterization of the association process; DMSO is a strong perturbant of proteins and peptides at higher concentrations due to the disruption of intramolecular hydrogen bonds. Hence at high concentrations, DMSO will act as denaturant, thereby dissociating GED assembly into 15 kDa monomers. These monomers are easily accessible to NMR for detailed characterization. Starting from the denatured monomeric state in 100% DMSO, we gradually decrease the concentration of DMSO by dilution with water, and in the end this closely approaches a lipid-aqueous environment. So by monitoring the hierarchy of association and folding of GED by DMSO dilution, we have derived valuable insights into the assembly process of the protein which, in turn, have a telling implication for the oligomerisation of dynamin.

## Results and Discussion

### 1. Gradual disappearance of peaks in the HSQC spectra with DMSO dilution

**Figure 1:**
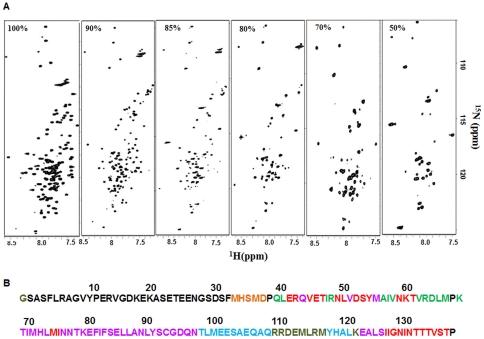
Summary of the gradual disappearance of the peaks with decreasing DMSO-d6 concentrations. (**A**) ^1^H-^15^N HSQC spectra of GED in 100%, 90%, 85%, 80%, 70% and 50% DMSO-d6 at 45°C, as marked in the left hand panel within the spectra. (**B**) The additional residues disappearing in the HSQC spectra with decreasing DMSO concentrations are color coded on the primary sequence of GED as: 100% (olive), 90% (cyan), 85% (pink), 80% (red), 70% (green) and 50% (orange).

On monitoring the Heteronuclear Single Quantum Coherence (HSQC) spectra at different DMSO concentrations, we found that there is a gradual disappearance of peaks with decrease in concentration of the denaturant (**Figure 1A**). This indicates that there is a gradual segment-wise association of the dissociated units into oligomers and the exchange processes encompass progressively larger number of residues. Peak assignments in all these spectra were obtained using the same procedures as used for 100% DMSO [12]. 124 of the expected 134 non-Proline residues are present in the HSQC spectrum [12] of GED in 100% DMSO. At 90% DMSO, the peaks corresponding to the stretch Thr98 - Gln106 disappear along with the segment Tyr117- Leu120 in the C-terminal end, resulting in the appearance of a total of 108 peaks in the spectrum. The peaks corresponding to the two contiguous stretches Thr69 – Asn97 and Glu122 – Ser125 disappear along with Gln43, Val51 and Met55 at 85% DMSO. On dilution of DMSO further to 80%, the stretch Ile126 - Thr137 present in the C-terminal end disappears along with few discrete residues in the N-terminal half of the polypeptide chain like Glu41- Arg42, Val44 - Thr46, Asn49 - Leu50, Asp52 – Tyr54, Asn59 – Thr61 and Met74 – Ile75, thus resulting in only 51 peaks in the HSQC spectrum. In 70% DMSO, 36 non Proline peaks were present in the spectrum and the disappeared peaks belong to the segments Gln39 – Leu40, Ile47 – Arg48, Ala56 – Val58, and Val62 – Lys68. We carried the dilution of DMSO till 50%; at this condition the HSQC spectrum contains 30 peaks which belong to the flexible N-terminal segment, Ser2 – Phe32. These peaks almost correspond to the peaks as in native GED excepting for the stretch Leu5 – Gly9 [13] which is absent in the HSQC spectrum of the latter. The peaks disappearing at different DMSO concentrations have been summarized in **Figure 1B**, where the peaks vanishing at 100%, 90%, 85%, 80%, 70% and 50% DMSO have been marked with olive, cyan, pink, red, green and orange on the primary sequence of the protein.

**Figure 2:**
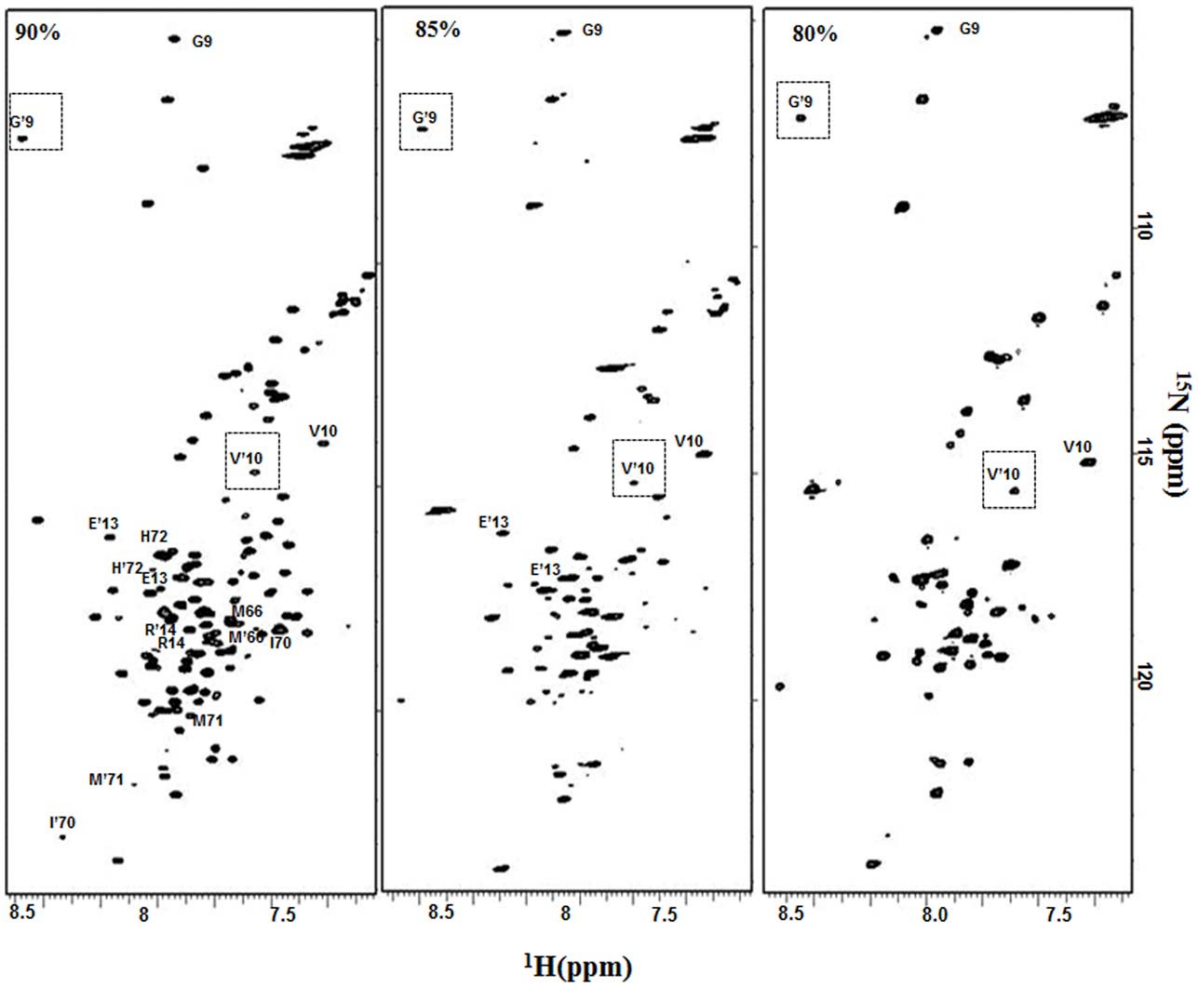
Slow conformational exchange in the equilibrium states. Illustrative representation of the residues showing two sets of peaks in the HSQC spectra at various DMSO dilutions, as indicated at the top left corner of the spectrum. The alternate sets of peaks in the HSQC spectra for the residues are indicated by primed and unprimed annotations. The boxed primed residues in the HSQC spectra indicate the similar positions of the alternate sets of peaks at various DMSO concentrations.

It is important to point out here that the HSQC spectra of GED at varying concentrations of DMSO show some additional peaks of low intensity. These arise due to the presence of more than one species stabilized in the DMSO denatured ensembles which are in slow exchange with each other. These were identified on the basis of observed sequential connectivities to neighboring residues. In 100% DMSO, Gly9, Val10, Glu13, Arg14, Val15, Lys18, Asp37, Gln39, Arg42, Asn49, Lys68, Ile70, Met71, Leu73, Met74, Leu86, Ser136 and Thr137 show two sets of cross peaks [14]. Similarly, in 90% DMSO, certain residues in the stretches Ser2 - Leu6, Gly9 – Tyr11, Glu13- Arg 14, Ser29 – Asp30, Met66 and Ile70- His72 exhibit slow conformational exchange in the chemical shift timescale. The connectivities between the residues attaining alternate conformations in the stretch Ile70- His72 in CBCANH experiment is shown in **Figure S1**. In 85% DMSO, the stretches Phe5 – Leu6, Gly9 – Tyr11, Glu13- Arg14 and Asn49 while in 80% DMSO, Ser2 – Leu6 and Gly9 – Val10 show two sets of cross peaks. Some of the residues showing two sets of peaks in the HSQC spectra at various DMSO dilutions have been illustrated in **Figure 2**. In order to ascertain whether the observed heterogeneity giving rise to the minor sets of peaks arise from cis-trans isomerism around a peptide bond preceding a Proline, we recorded a 3D (H)C(CH)(CO)NH TOCSY experiment of GED in 100% DMSO and chemical shift analysis of Cβ and Cγ resonances of the 3 Proline (Pro 12, 38, 67) residues (arising from both conformations) indicate trans conformation of the peptide bonds in all of them, since the difference in the chemical shifts of Cβ and Cγ was found to be ∼5 ppm (**Figure S2**). Since the minor sets of peaks arising from heterogeneous conformers are present almost at similar positions in the respective HSQCs (**Figure 2**), we extend our observation to assume that the peptide bond preceding the Pro residues remain in trans conformation in various DMSO-dilutions. The transverse relaxation rates of these alternate states are also distinctly different, reflective of the differential flexibility of these segments in the two conformations. This indicates that these conformations may arise due to the exchange between the monomer and oligomer, or between unstructured and structured forms.

### 2. Structural characteristics of the equilibrium states at different DMSO concentrations

**Figure 3:**
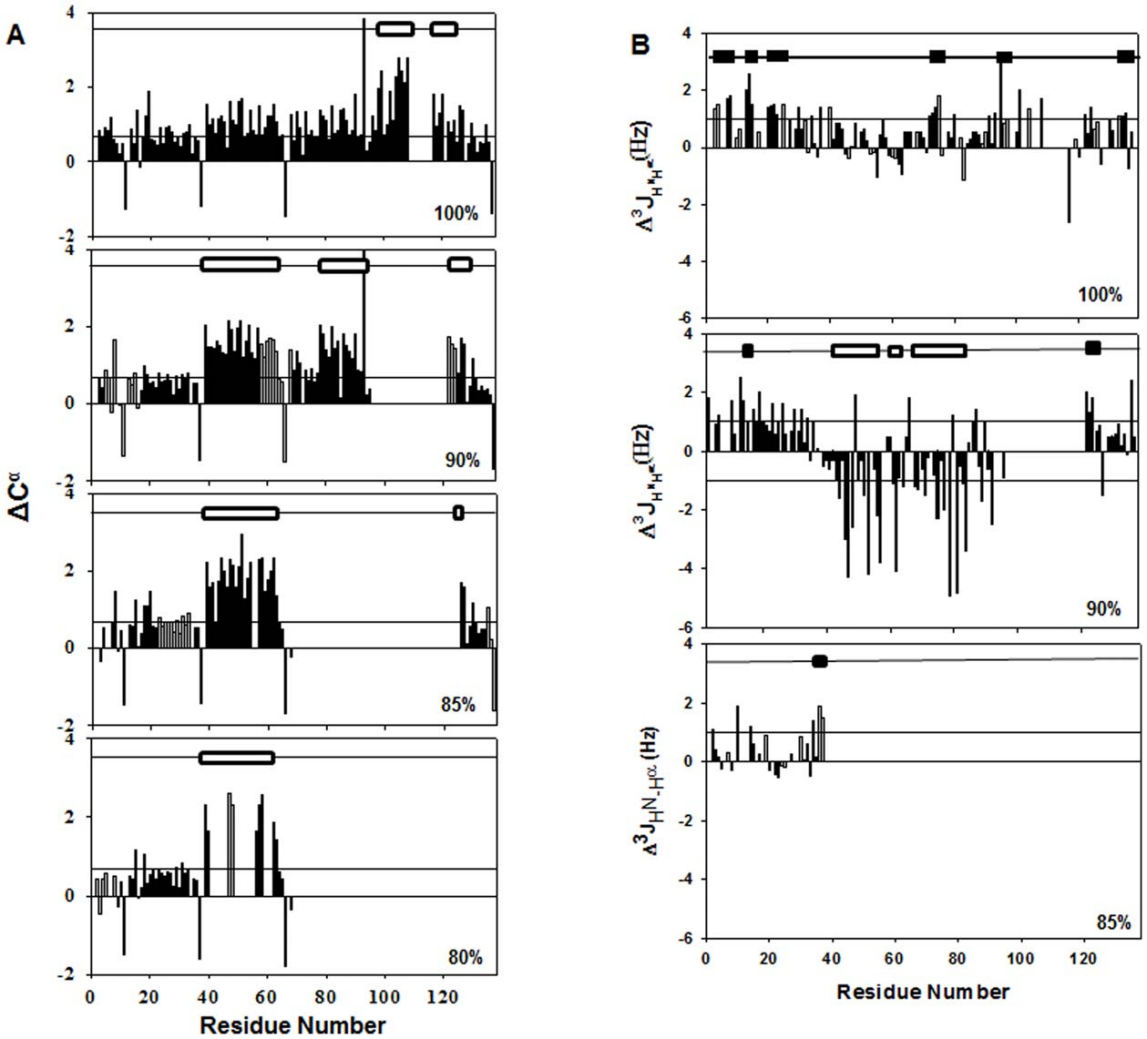
Structural propensities of the equilibrium states of GED with DMSO–d6 dilution. (**A**) Residue-wise plots of C^α^ secondary chemical shifts of GED in 100%, 90%, 85% and 80% DMSO-d6 at 45°C. Positive deviations of C^α^ indicate alpha helical propensities whereas negative deviations indicate beta preferences. The deviations of C^α^ chemical shifts at different DMSO concentrations were measured by using the formula, ΔC^α^ = C_obs_−(p1*C_ran,dmso_+p2*C_ran,aq_) ,where p1 and p2 are the relative contributions of random coil chemical shifts in DMSO and aqueous environment, respectively. The straight line in the plots is drawn at 0.7 ppm to indicate the uncertainty in the spread of C^α^ chemical shifts. (**B**) Residue wise plots of the secondary ^3^JH^N^_-_H^α^ coupling constants of GED in 100%, 90% and 85% DMSO-d6. At 85% DMSO, the splittings of only the N-terminal residues could be measured. The high-resolution HSQCs of GED in lower concentrations of DMSO did not show any measurable splitting. The empty and the filled black bars at the top of the graphs represent the helical and extended beta sheet like propensities.

The residual structures in denatured proteins implicate interactions involved in very early stages of protein folding and dictate the trajectory of folding. The chemical shift values of the amino acids are conventionally used to predict the structural propensities of proteins in aqueous solvents [15], [16], [17], [18]. Deviations of C^α^ chemical shifts from random coil values are used to obtain secondary structural information along the protein backbone [18]. Positive deviation of C^α^ indicates alpha helical preferences while a negative deviation is suggestive of β-sheet like propensities. However, chemical shift values are largely environment dependent and hence are largely influenced by solvent conditions. DMSO is an aprotic solvent and the random coil C^α^ chemical shift values of the amino acids in DMSO [19] are substantially different from those in aqueous environment [20]. In order to get a qualitative insight into the structural characteristics of the different equilibrium states of GED, we calculated the deviations by using the formula ΔC^α^ = C_obs_−(p1*C_ran,dmso_+p2*C_ran,aq_), where p1 and p2 are the weighted contributions of random-coil chemical shifts in DMSO (C_ran,dmso_) and aqueous (C_ran,aq_) environments, respectively, as determined from the relative exposure of the protein to the two solvents. We are aware that such a calculation may not truly represent the random coil chemical shifts at that solvent environment, since the affinities of the short peptides used for determination of random coil values for the two solvents may not be identical. Likewise, the affinities of different segments of the chain in the unfolded state for the two solvents also may not be the same; we have no information about these, and this may render quantitative interpretations on residual structures inappropriate. However, we believe that the present approximation may still serve to gain insights into qualitative trends as the conditions are changed. We only considered the contiguous stretches of residues which show deviations significantly higher than the uncertainty in the spread of the C^α^ chemical shifts (∼0.7 ppm) to infer about structural propensities. The residue-wise ΔC^α^ for the equilibrium states of GED in 80, 85, 90 and 100% DMSO are plotted in **Figure 3A**. As is evident from the figure, in 100% DMSO, though the polypeptide chain exhibits small positive deviations in certain segments of the polypeptide chain, the effect is most pronounced for the stretch, Ala105 – Leu120, indicating presence of helical propensities in this segment. With decrease in the concentration of DMSO from 100% till 85%, there is a gradual increase in the amount of positive deviation in different segments of the polypeptide chain, except the N-terminal 40 residues where the chemical deviations are relatively small and not interpretable. This indicates an increase in the helical content and an approach towards a more ordered structure. For a more reliable estimation of the structural preferences, we calculated the three bond coupling constants, ^3^JH^N^-H^α^ from high resolution HSQCs; these are highly sensitive to the backbone torsion angle (Φ) and are largely independent of the solvent condition. The J values range from 3–5 Hz for α-helix, 8–11 Hz for β sheeted structure and 6–8 Hz for a random coil. Depending on the preceding residue (aromatic/non-aromatic side chains) the J_random_ values for different residues were corrected [21]. Negative deviations from random coil values indicate helical propensities (this also includes PPII structures) while a positive deviation is indicative of β-sheet propensity. In 100% DMSO, the J_obs_ - J_random_ indicate β propensities in the N terminal regions while helical propensities in certain segments of the C-terminal half, whereas the residues corresponding to the theoretical N-terminal helix showed mostly random coil like propensities [12] (**Figure 3B**). It is interesting to note that with change of concentration of DMSO from 100% to 90%, the structural propensities in the segment Gln43 - Ser92 change largely from random coil and β-sheet [12] to α-helical. In 90% DMSO, the coupling constants of 94 out of 108 assigned residues were calculated using J_obs_ - J_random_ values. These deviations ranged from +2.5 to −4.9 Hz as demonstrated in **Figure 3B**. An illustrative example of the quality of splittings in the high-resolution HSQC at 90% DMSO is provided in **Figure S3**. The flexible N terminal and the C terminal edge of the second alpha helix (this refers to the secondary structural elements in native GED as predicted by Expasy proteomics tool) show positive deviations indicating β propensities in these segments. The segment Gln43-Ser92 shows large negative deviations indicating helical propensities. In 85% DMSO, the splittings of only 27 peaks could be measured; these residues belong to the flexible N-terminal end. The remaining residues did not show measurable splitting indicating presence of helical structure/structural propensities in those segments. In 80% DMSO, however, the high resolution HSQC spectra of GED did not yield measurable splitting. The gradual decrease in the coupling constants leading finally to the absence of splittings with DMSO dilution indicate a possibility of helicity getting progressively induced in the polypeptide chain, which is further consolidated by the observed trends in secondary C^α^ chemical shifts. We also mention here that in cases of observed discrepancies in the structural preferences derived from chemical shift deviations and coupling constants in certain segments of the protein chain (like Glu122- Ile127 in 90% DMSO), the latter serves as a more reliable indicator. Based on the above observations, we assume that with decrease in concentration of DMSO, the polypeptide chain gradually tends to lose extended random coil like topologies and attains native-like structures. As this leads to association, it is evident that association of the oligomer is intimately related to the folding of the monomer.

### 3. Motional characteristics along the chain in the equilibrium states of GED at different DMSO concentrations

**Figure 4:**
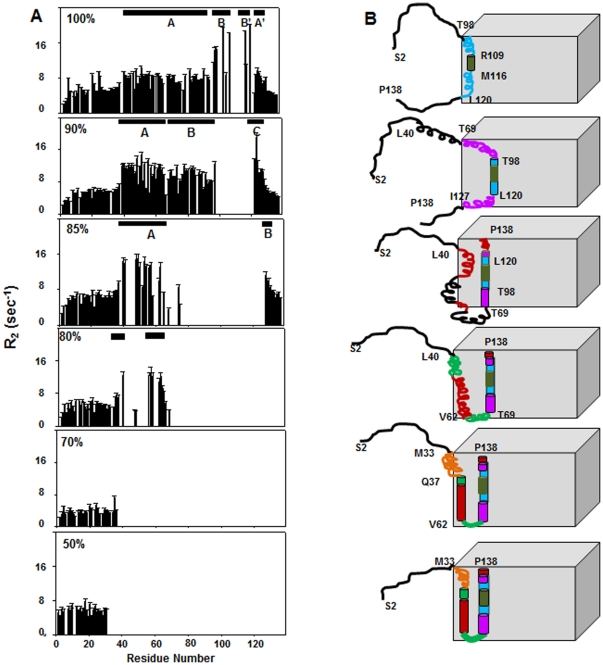
Motional characteristics of the equilibrium states of GED and hierarchy of association with DMSO–d6 dilution. (**A**) ^15^N transverse relaxation rates (R_2_) measured at the corresponding DMSO concentrations marked on the top left side of the graphs. The domains (see text) of slow motions due to conformational exchange are marked with black bars on top of the graphs. The residues having R_2_ values higher than the remaining segments at a particular DMSO concentration disappear from the HSQC spectrum at the next DMSO concentration. (**B**) The 3D cartoon representation of the hierarchy of association of GED chain with DMSO dilution. The cube represents the core and if a chain segment is outside the cube, its proximity to the cube is loosely inversely related to the extent of burial inside the assembly. The helices are shown by cylinders and the segments undergoing conformational exchange are denoted by compressed spring like representation. The segments have been color coded following the same convention as mentioned in Figure 1(B). The geometric arrangement is purely arbitrary and hypothesis-driven. In 100% DMSO, the polypeptide chain is denatured. The absence of the peaks corresponding to the stretch Arg109-Met116 flanked by two stretches Asn97- Gln108 and Tyr117- Leu120 of high R_2_ values indicates that this segment is undergoing slow conformational exchange and it may act as the nucleation site of folding and aggregation. In 90% DMSO, the formation of C-terminal helix is then extended and covers the stretch from Thr98 – Lys121. Helix formation extends and includes the stretches of residues Thr69 – Asn97 and Tyr117- Leu120. At 85% and 80% DMSO, though transient helix formation sets in the stretch of residues Leu40 – Lys68, this part is not tightly packed in the core and remains somewhat flexible compared to the C-terminal helix. Proline 67 may act as a flexible kink around which the polypeptide chain bends and flips in and out of the assembly. Finally at 50% DMSO, both the helices are completely formed.

The transverse relaxation rates, R_2_, obtained from Carr Purcell Meiboom Gill (CPMG)-based transverse relaxation experiments are largely sensitive to motions on ms-µs time scale and have contributions from both intrinsic relaxation rates and exchange contributions, R_ex_. R_2_ provides a good probe to monitor the changes in the regions of conformational exchange in a protein as it folds. So R_2_ values were measured for the assigned residues at every DMSO concentration (as shown in **Figure 4A**), which provide insight into the changes in the regions of conformational exchange as the protein undergoes folding and concomitant association. In 100% DMSO, the polypeptide chain shows 4 regions of high R_2_, namely A (Leu40 - Tyr91), A′ (Leu124 - Ile130); B (Asn97 - Gln108), B′ (Tyr117 - Leu120) flanking the segment Arg109-Met116 [12]. In 90% DMSO, out of the 108 assigned residues the dynamics of only 105 residues could be studied on the 800 MHz instrument, due to overlap of peaks in the middle region of the spectrum. As is evident from **Figure 4A**, considerable variations were observed in the transverse relaxation rates which varied from 2.2±0.2–19.1±1.3 s^−1^ (± indicates standard error). Such differences may arise due to differences in short-range interactions and topological fluctuations along the polypeptide chain. The higher R_2_ values in the segment Leu40–Asn97 may be attributed to conformational exchange of the dissociated flexible chain with the structured form. This is again consolidated by the presence of alpha helical propensities in this segment. From the trend of the spread in R_2_ values, three regions of slow motions, namely A (Glu39-Leu65), B (Ile75-Asn97) and C (Glu122-Ile128) were identified. These segments are not completely flexible due to the involvement of their adjacent segments in the core or they may be undergoing structural transitions. In 85% DMSO, the R_2_ values were measured for 62 out of 67 assigned residues due to the overlap of few peaks. The values covered a wide range from 2.61±0.10 s^−1^ to 15.02±1.10 s^−1^ with an average value of 7.3±0.35 s^−1^. High R_2_ values due to conformational exchange were observed for the stretches Asp37- Glu41, Arg48 – Leu50, Ser53 – Ile57, Arg63 – Asp64 and Gly128 – Thr133. In 80% DMSO, however, discrete stretches of residues in the N-terminal half of theoretical helix 1 show higher R_2_ values compared to the N-terminal end which reflect upon certain degree of restriction in the flexibility of these segments. We were able to measure the dynamics of 46 out of 48 assigned residues present in the HSQC spectrum. As seen in **Figure 4A**, the R_2_ values range between 2.03±0.02 s^−1^ to 13.21±0.12 s^−1^ (average being 5.99 s^−1^); the residues with high R_2_ being concentrated in the stretches Ser35-Leu40 and Ala56–Leu65. The increase in R_2_ values of these stretches can be attributed to the exchange between helical and unstructured forms as well as transiently buried and exposed forms. This also reflects on the loose packing of this segment in the core of the assembly compared to the C-terminal half. In 70% DMSO, the peaks present in the HSQC spectrum belonging to the N–terminal end, till Met36, showed more or less uniform R_2_ values owing to their similar level of flexibility. In 50% DMSO, however, the higher values of R_2_ indicate that the N-terminal end is less flexible than in 70% DMSO due to the association of the adjacent segment Met33 – Met36 into the assembly. As is observed from the structural data, with dilution of DMSO, the polypeptide chain tends towards a more structured form and the segments which show structural (mostly helical) propensities show high R_2_. This leads us to assume that the increase in values of R_2_ is possibly due to intermediate time scale exchange between the partially structured and unstructured forms of the protein. Moreover, the gradual disappearance of the peaks showing high R_2_ values at a particular DMSO concentration, on the next dilution, which is due to their inclusion in the core of the aggregate, supports our model of folding and concomitant association.

### 4. Hierarchy of self association along the chain in GED – mechanistic insights

The intrinsic properties of the different equilibrium states of GED under varying concentrations of DMSO have led to an insight into the hierarchy of inclusion of different segments of the polypeptide chain in the assembly as the monomers begin to associate. This has been summarized in **Figure 4B**. Structural and motional characterization of the equilibrium states revealed that gradual aggregation of the monomer into oligomers is preceded by helix formation and the mode of association would be dictated by the surface properties of the helices. With dilution of the denaturant we see an increase in the residual structural propensities and a gradual disappearance of peaks in the HSQC spectra. In 100% DMSO denatured state, the presence of four regions of high conformational exchange on ms-µs time scale, namely, domains A (Leu40 – Tyr91), A′ (Leu124- Ile130); B (Asn97- Gln108), B′ (Tyr117- Leu120), and the stretch of missing residues (Arg109 - Met116) flanked by the stretches B and B′ indicate that this portion of the polypeptide chain may act as the potential nucleation site for aggregation and structure formation on further dilution of DMSO. A careful examination reveals that the segment (Arg109 - Met116) has a stretch of adjacent polar amino acids with charge complementarity. Arg109 has a high probability of interacting with Glu112 and similar attractive interaction is also possible between Glu112 and Arg115. This sort of attractive intramolecular interaction may result in H-bond formation which could drive helix formation. The positive charge of the side-chains of the basic amino acid Arginine can also participate in some attractive intermolecular interaction with the negatively charged acidic side chains of Glutamic acid and Aspartic acid resulting in transient electrostatic clustering. These charge interactions are stabilized by certain orientations of the side chains thereby facilitating folding and association. Also, the high abundance of Alanines and Leucines which are efficient helix inducers in the segment further assist the formation of helices, provide a hydrophobic surface and thereby lead to self association and inclusion of this segment in the assembly. This prediction gains support from the fact that in 90% DMSO, the continuous stretch of residues, Thr98–Lys121, which coincides with B and B′ stretches in 100% DMSO having high R_2_ values disappear. Thus at 90% DMSO the folding and association elongates in both the directions around the nucleation site. The newly formed helices are probably stacked together to form cylinders and this sort of arrangement facilitates the stacking of maximum oligomer building units. It is expected that the three stretches of residues with high R_2_, A (Gln39-Asp64), B (Thr69-Asn97) and C (Ala123-Ile128) are more probable to go into the core of the aggregate with simultaneous helix formation on further dilution. That is exactly what we observe in 85% DMSO concentration, where the disappeared residues belong to a contiguous stretch (Thr69 - Ser125) in the C-terminal half and one discontinuous (Gln43 - Lys68) stretch in the N terminal half. So it may be assumed that the helix extends further as a continuation of a single long structural unit till Thr69 and N-terminal helix formation sets in the segment (Gln43 - Met66) concomitantly with the inclusion of these nascent helices in the assemblies. In 80% DMSO, few more residues in the stretch Glu41-Val51 and Ile126 –Thr137 go into the assembly. This observed hierarchy in the inclusion of different segments inside the assembly indicates that the C-terminal half of the protein is more prone to core formation and hence is more tightly packed compared to the N-terminal half. This assumption goes hand in hand with the hierarchy of solvent exposure as observed in the native state [12]. The presence of the peaks corresponding to the segment Val62 – Lys 68 in 80% DMSO flanked by the adjacent segments which have already disappeared indicates that Proline 67 may act as a flexible hinge, thereby transiently exposing the immediate neighboring residues while helix formation and association occur in the adjacent segments. This assumption can be consolidated by the fact that prolines are often found in hairpins and are known to cause kinking. Further, on decreasing the DMSO concentration to 70%, the entire segment of GED excepting the flexible N-terminal goes into the assembly. In 50% DMSO, the stretch Met33 - Asp37 also joins in the assembly formation while the extreme N-terminal residues remain flexible, which is what is seen in the native oligomer.

### 5. Model of GED self-association

**Figure 5:**
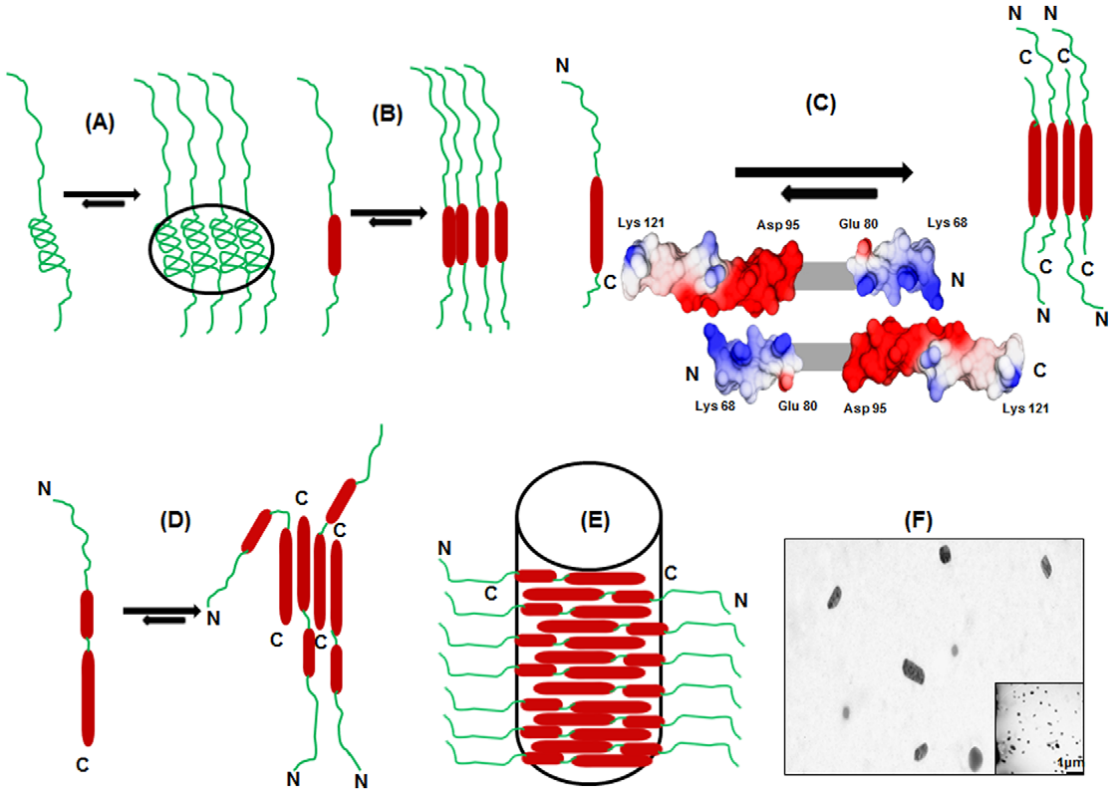
A possible model for concomitant folding of the GED chain and association by DMSO dilution. (**A**) The polar amino acid rich segment of the completely or partially dissociated polypeptide chain of GED monomer have high probability of helix formation due to the electrostatic interaction. The monomeric units come in close proximity of other similar units in the system due to intermolecular charge interactions thereby forming clusters. (**B**) Helix formation initiates in a certain segment of the C-terminal of the monomer and the nascent helices of the monomers stack together to form barrel like structures. (**C**) The helix extends in the neighboring segments. The vacuum electrostatic potential surfaces generated by NOC software (http://noch.sourceforge.net/) depict predominance of opposite charges in the segments Lys68 – Glu80 and Asp95 – Lys121, which favors anti-parallel organization of the newly formed helices of the monomeric units to form extended oligomeric units. (**D**) Helix formation initiates in the N-terminal half of the monomer. However Proline 67 acts as a kink around which the polypeptide chain can bend thereby providing certain degree of flexibility to the N-terminal half. These units simultaneously arrange themselves in a particular orientation such that the N-terminal helices of the monomeric units are not completely embedded in the core of the assembly; rather it has certain degree of flexibility. (**E**) The N-terminal helix is completely formed and the oligomers arrange themselves linearly in random numbers to form the extended oligomers with a wide diversity of size. (**F**) Enlarged view of a part of the TEM image of copper plate with ∼1 µM GED in water (inset) at room temperature. The image shows differential size and shape distribution of GED oligomers, although the majority show elongated structures. The concentration of the protein mentioned is not the true concentration due to the evaporation of water during sample preparation.

While the above analysis provides an insight into the step-wise participation of segments along the sequence in self-association, the actual model of association would be dictated by the surface properties of the helices; helices can be organized in parallel or antiparallel fashion. In the process of association the solvent is assumed to be discarded from the immediate vicinity of the aggregating units which directs the system towards a more compact and stabilized assembly formation. So the electrostatic characteristics of these surfaces may well be represented by the vacuum electrostatics. The vacuum electrostatic potential surface of the C-terminal helix indicates presence of opposite charges in the two stretches Lys68 – Glu80 and Asp95 – Lys121 (as depicted in **Figure 5C**), thereby favoring an antiparallel organization of the monomeric units which facilitates the proximity of these oppositely charged surfaces. Depending on the above observations, the step wise association and one particular model that can be derived for the assembly of GED is schematically depicted in **Figure 5 (A–E**). The linear stacking of the building blocks of the oligomer allows the N-terminal ends of all the polypeptide chain to be exposed to the solvent providing similar flexibility to all of them. This assumption is further supported by the fact that the flexible N-terminal residues give fairly intense signals in the HSQC spectrum and their R_2_ values remain similar throughout the segment. Most importantly, such an arrangement of monomeric units of GED shows fair possibility of giving rise to an elongated oligomer as observed in the TEM image (**Figure 5F**).

### 6. Relevance to dynamin self-assembly

**Figure 6:**
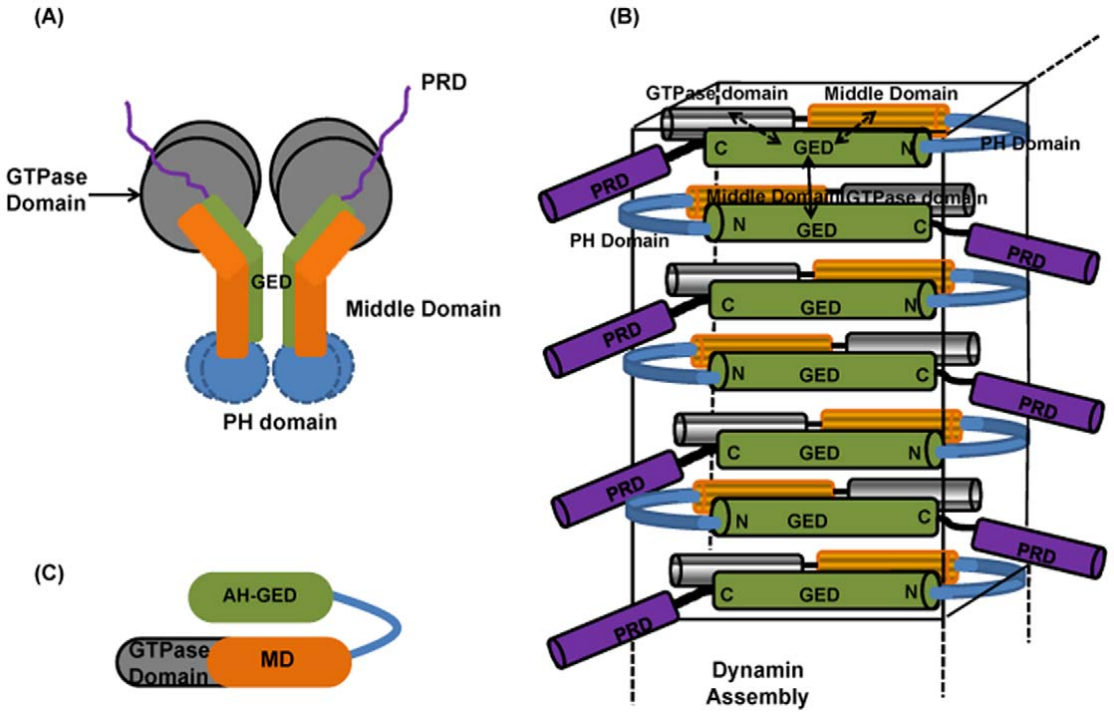
Models of dynamin assembly. (**A**) Schematic representation of the T-shaped dynamin tetramers or ‘dimer of dimers’ [26], [27] which explains the inter-domain interactions between the GED, middle Domain and GTPase domain. (**B**) A hypothetical model derived from the present observations for formation of an elongated oligomer of dynamin. The 4 cylinders of different colors indicate 4 different domains of dynamin: GTPase domain, Middle domain, GED and Proline – Arginine rich domain. Pleckstrin Homology domain is indicated by a twisted sheet. The arrangement of the dynamin monomers is such that the GED domains are antiparallel to each other. The reported interactions of GTPase domain and Middle domain with GED and self interaction of GED are denoted by double headed arrows (solid line for intermolecular interactions whereas dashed line for intramolecular interactions). The entire assembly is fitted inside a box to provide a 3D view of the molecules. The extended dashed lines in different direction indicate that the assembly can extend both vertically and horizontally. (**C**) Representation of intramolecular back-folding of the GED domain onto the GTP-middle domain as suggested by Zhu et al (2004) which is well in accordance with our model.

Dynamin self-assembles to form ordered tubes in presence of negatively charged liposomes and forms extended spiral structure around the neck of vesicles [22], [23], [24], [25]. The rings of the spiral generally consist of ∼30 dynamin molecules. The mechanism of dynamin assembly during endocytosis has remained a topic of considerable interest and various researchers have provided insights into the interactions of the different domains responsible for dynamin oligomerization based on their extensive studies on full length/individual domains of dynamin, on various mutants of the protein, as well as on other dynamin-like proteins [9], [10], [26], [27], [28]. The models proposed, though based on extrapolation of their experimental observations, are to a fair extent speculative. However, regardless of the model, it has been agreed univocally that the middle domain (MD) and the GTPase Effector Domain (GED) are responsible for formation of the higher order structures [10], [28] in dynamin, and interaction between GTPase monomers are dependent upon assembly promoted by these two domains. These domains form a stalk in the T-shaped dynamin and present an assembly interface on opposites faces of the dimer stalk that mediate intermolecular interactions along the radial axis of the protein [29] (**Figure 6A**). GED has been shown to interact strongly with the GTPase domain [8], [23] and removal of GED eliminated dynamin-dynamin interactions [10]. Further, GED binds to itself forming homodimers [9] or tetramers [10] as well as to the middle domain (residues 320–349) [9]. However, the structural details of the assembly process involving complete GED segment at residue level are not clearly understood yet. Hence the characteristics of full length GED with regard to its association properties have immense potential to add further insights into the organization of the dynamin assembly.

**Figure 7:**
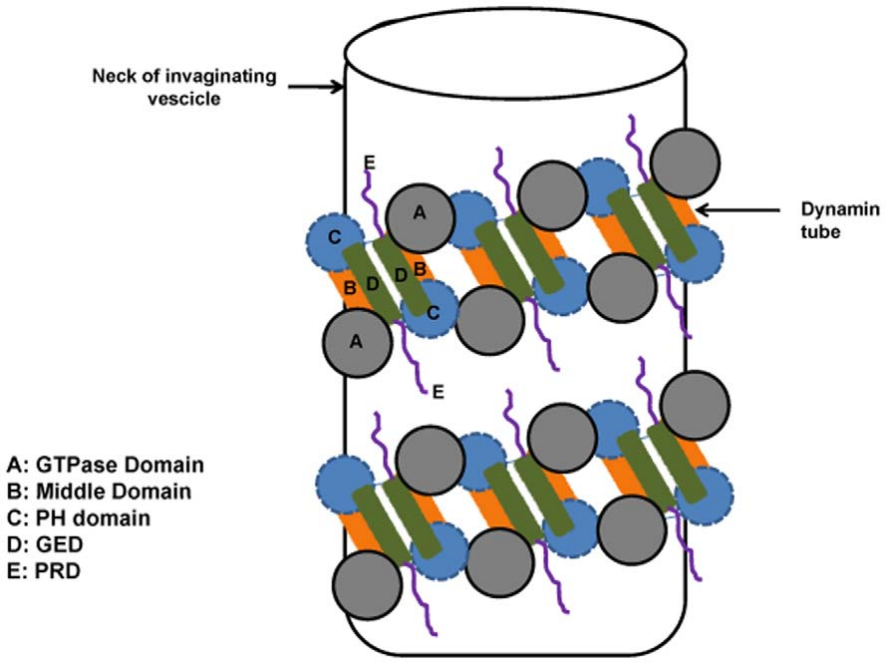
Dynamin tubes around the neck of invaginating vesicles. Schematic representation of the organization of dynamin assembly wrapped around the neck of invaginating vesicles of parent membrane. The anti-parallel organization of the GED units results in a zigzag orientation of dynamin units which favor efficient binding of the dynamin tube around the neck of the vesicles. The PH domain binds to the lipid vesicles forming the base followed by GED and Middle domain forming a neck from which the GTPase domain protrudes as a head.

In this background, we try to correlate the results obtained in our study to gain useful insights into one of the possible mechanism of association and topology of self assembled dynamin in presence of lipid-water environment. In the light of the observations on GED, we assume that the association of dynamin may be driven by some conformational rearrangement in the protein which brings the GED units in close proximity, thereby stabilizing the charge interactions leading to a more organized system. The highly hydrophobic surfaces on the GED units then try to exclude solvent from their immediate vicinity. The GED domain folds back on the middle domain to favor intramolecular interactions resulting in a compact arrangement of the units in the oligomer. The intermolecular interactions between the GED molecules favor further association to form larger oligomers. Taking into consideration the reported interactions between the different domains in dynamin and our results on GED, favoring anti-parallel organization we speculate a model of elongated dynamin oligomer as depicted in **Figure 6B**. This model goes in accordance with the study on human-dynamin like GTPase Drp1, where intra-molecular back-folding of the GED domain onto the GTP-middle domain has been demonstrated [26] (**Figure 6C**). The favored intra-molecular interactions of GED and GTP-middle domain as well as intermolecular interaction of GED domains with other GED domains are also incorporated in this model. Though Okomato et al suggest parallel orientations of the individual dynamins in their tetramer model, our results with regard to GED suggest that an anti-parallel arrangement is also equally feasible in a long tube-like assembly, and in fact, this enables more efficient binding of the PH domains in the long dynamin spiral to the stem of the excising membrane vesicle (**Figure 7**). The proximity of the C-terminal end of GTPase domain to GED in our proposed model can possibly facilitate generation of mechanical energy at the long fibre on GTP hydrolysis required for vesicle scission and thereby supports Cryo-EM studies [30] that predict dramatic rearrangement and constriction in the GED and Middle Domains following GTP hydrolysis, resulting in scisson and rapid disassembly. The schematic arrangement of the domains depicted in our model is surely very simplistic and better insight would require high resolution structure determinations of the assembled species.

### Conclusion

With an aim to understand the process of association of ‘coiled coil’ GTPase Effector Domain (GED) which is the chief mediator of self-association of dynamin into spiral structures around lipidic vesicles during endocytosis, we have performed a detailed analysis of the structural and motional properties of the equilibrium states with DMSO dilution in order to achieve a lipid-water like environment. From the comprehensive investigation of the equilibrium states, it is apparent that in the process of association, the C-terminal helices from different GED molecules stack together tightly and linearly while the helix in the N-terminal end, though involved in the assembly, has certain degree of surface exposure and flexibility. The surface properties of the monomeric units favor anti-parallel organization of the building blocks in the assembly, and their relative organization point toward an extended topology. This sort of arrangement does not confine the number of monomeric units constituting the assembly which explains the wide diversity in size of the oligomers. The probable mode of association of GED in presence of lipid-water like environment, as proposed by us here, combined with the different inter and intra-domain interactions of dynamin serves as significant stepping stone in understanding the self- association process of the protein around the neck of invaginating vesicles during endocytosis. More elaborate studies using different mutants of GED would be required to shed greater light on the factors driving self-association of GED. Similarly, more data on inter-domain interaction would be required to throw light on the mechanism of dynamin self assembly.

## Materials and Methods

### Protein expression and purification

GED was expressed and purified as described elsewhere [31].

### NMR sample preparation

For NMR studies, isotopically labeled protein was prepared from M9 media by using (4 g/l) ^13^C glucose and (1 g/l) ^15^N NH_4_Cl as the sole sources of Carbon and Nitrogen, respectively. The purified protein was exchanged with 20 mM acetate buffer (pH 6) containing 1 mM EDTA, 150 mM NaCl, and 1 mM DTT and concentrated to 1.5 mM. Samples were flash-frozen in liquid nitrogen and lyophilized. The samples were equilibrated with different concentrations of denaturant (100%, 90%, 85%, 80%, 70% and 50% (vol/vol) DMSO-d6). Trifluoroacetic acid was added to the samples in order to maintain the apparent pH at 4.5 and the samples were allowed to come to equilibrium for 24 hours before the start of experiment. A series of HSQC spectra were recorded at different times while performing the experiments. The spectra remained unchanged throughout confirming that equilibrium had been attained before the start of the experiments.

### NMR data acquisition and processing

NMR experiments were performed on Bruker Avance (800 MHz) spectrometer at 45°C. The acquired data were processed using Felix (Accerlys Software Inc., San Diego, CA) and analyzed using Felix and CARA [32]. Backbone assignments of the polypeptide chain at DMSO concentrations of 80, 85, 90, 100% were obtained with the aid of heteronuclear triple resonance experiments, HNN [33], CBCANH, CBCA(CO)NH, HNCO and HN(CA)CO and TOCSY-HSQC. Since the resonances were easily transferrable in 70% and 50% DMSO, only TOCSY-HSQC was used to confirm the spin systems. DSS was used to reference the ^1^H, ^13^C and ^15^N chemical shifts in the standard way as done in case of aqueous solvents by monitoring the exact chemical shift of the water peak keeping DSS peak at 0 ppm. 3D (H)C(CH)(CO)NH TOCSY experiment was recorded with 1024×64×128 complex points along t3, t2 and t1 dimensions with spectral widths 9, 22 and 76 ppm, respectively. ^3^JH^N^-H^α^ coupling constants were measured from high resolution HSQC spectra which were recorded with 16384×1024 complex points with spectral widths of 9 and 24 ppm along the t2 and t1 dimensions respectively. The relaxation measurements were carried out using the pulse sequences described by Farrow et al. [34]. ^15^N transverse relaxation rates (R_2_) were measured with CPMG delays 10, 30, 50, 70, 90, 110, 130, 150, 170, 190 ms.

## Supporting Information

Figure S1 **The stretch of residues (I70-H72) exhibiting two sets of peaks due to slow conformational exchange are marked on the HSQC spectrum of GED in 90% DMSO at 45°C as primed and unprimed residues.** The sequential connectivities of the contiguous stretch of alternate residues (I′70-H′72) connected to T69 in the CBCANH experiment are shown. The red peaks indicate positive contour whereas green indicate negative contours. The arrows are connected between the centers of the peaks to indicate the connectivities between C^α^ and C^β^ of i and i+1^th^ residue.
(TIF)

Figure S2 Illustrative strips from (H)C(CH)CONH TOCSY spectra of GED in 100% DMSO-d6 showing the ^13^C chemical shifts of (A) Pro 12′ (B) Pro 12 and (C) Pro 67. The chemical shift differences between the ^13^Cβ and ^13^Cγ nuclei are ∼5 ppm indicating trans conformation of the peptide bonds in the heterogeneous ensemble.
(TIF)

Figure S3 **Selected region of the high resolution ^1^H-^15^N HSQC of GED at 90% DMSO to show the quality of the spectral resolution.** Splitting in the peaks was used to measure the ^3^JH^N^-H^α^ coupling constants.
(TIF)

## References

[1] Dobson CM (1999). Protein misfolding, evolution and disease.. Trend Biochem Sci.

[2] Dobson CM (2006). Protein aggregation and its consequences for human disease.. Prot Pept Lett.

[3] Jaenicke R, Lilie H (2000). Folding and association of oligomeric and multimeric proteins.. Adv Prot Chem.

[4] Conner SD, Schmid SL (2003). Regulated portals of entry into the cell.. Nature.

[5] Praefcke GJ, McMahon HT (2004). The dynamin superfamily: universal membrane tubulation and fission molecules?. Nat Rev Mol Cell Biol.

[6] Tuma PL, Stachniak MC, Collins CA (1993). Activation of dynamin GTPase by acidic phospholipids and endogenous rat brain vesicles.. J Biol Chem.

[7] Muhlberg AB, Warnock DE, Schmid SL (1997). Domain structure and intramolecular regulation of dynamin GTPase.. Embo J.

[8] Sever S, Muhlberg AB, Schmid SL (1999). Impairment of dynamin's GAP domain stimulates receptor-mediated endocytosis.. Nature.

[9] Smirnova E, Shurland DL, Newman-Smith ED, Pishvaee B, van der Bliek AM (1999). A model for dynamin self-assembly based on binding between three different protein domains.. J Biol Chem.

[10] Okamoto PM, Tripet B, Litowski J, Hodges RS, Vallee RB (1999). Multiple distinct coiled-coils are involved in dynamin self-assembly.. J Biol Chem.

[11] Chugh J, Chatterjee A, Kumar A, Mishra RK, Mittal R (2006). Structural characterization of the large soluble oligomers of the GTPase effector domain of dynamin.. Febs J.

[12] Chakraborty S, Hosur RV (2011). NMR Insights into the Core of GED Assembly by H/D Exchange Coupled with DMSO Dissociation and Analysis of the Denatured State.. J Mol Biol.

[13] Chugh J, Sharma S, Hosur RV (2008). NMR insights into a megadalton-size protein self-assembly.. Prot Sci.

[14] Chakraborty S, Hosur RV (2011). Resonance assignments of GTPase effector domain of dynamin in the aprotic solvent deuterated dimethyl sulfoxide.. Biomol NMR Assign.

[15] Wishart DS, Bigam CG, Holm A, Hodges RS, Sykes BD (1995). H-1, C-13 and N-15 Random Coil NMR Chemical-Shifts of the Common Amino-Acids .1. Investigations of Nearest-Neighbor Effects.. J Biomol NMR.

[16] Wishart DS, Sykes BD (1994). Chemical-Shifts as a Tool for Structure Determination.. Nucl Magn Reson.

[17] Wishart DS, Sykes BD (1994). The C-13 Chemical-Shift Index - a Simple Method for the Identification of Protein Secondary Structure Using C-13 Chemical-Shift Data.. J Biomol NMR.

[18] Wishart DS, Sykes BD, Richards FM (1991). Relationship between Nuclear-Magnetic-Resonance Chemical-Shift and protein secondary structure.. J Mol Biol.

[19] Grathwohl C, Wuthrich K (1974). Carbon-13 NMR of the protected tetrapeptide TFA-Gly-Gly-L-X-L-Ala-OCH_3_, where X stands for the 20 common amino acids.. J Magn Reson.

[20] Richarz R, Kurt W (1978). Carbon-13 NMR chemical shifts of the common amino acid residues measured in aqueous solutions of the linear tetrapeptides H-Gly-Gly- X-L-Ala-OH.. Biopolymers.

[21] Penkett CJ, Redfield C, Dodd I, Hubbard J, McBay DL (1997). NMR analysis of main-chain conformational preferences in an unfolded fibronectin-binding protein.. J Mol Biol.

[22] Carr JF, Hinshaw JE (1997). Dynamin assembles into spirals under physiological salt conditions upon the addition of GDP and gamma-phosphate analogues.. J Biol Chem.

[23] Hinshaw JE (1999). Dynamin spirals.. Curr Opi Struc Biol.

[24] Hinshaw JE, Schmid SL (1995). Dynamin Self-Assembles into Rings Suggesting a Mechanism for Coated Vesicle Budding.. Nature.

[25] Muhlberg AB, Schmid SL (2000). Domain structure and function of dynamin probed by limited proteolysis.. Methods.

[26] Blackstone C, Zhu PP, Patterson A, Stadler J, Seeburg DP (2004). Intra- and intermolecular domain interactions of the C-terminal GTPase effector domain of the multimeric dynamin-like GTPase Drp1.. J Biol Chem.

[27] Schmid SL, Chappie JS, Acharya S, Leonard M, Dyda F (2010). G domain dimerization controls dynamin's assembly-stimulated GTPase activity.. Nature.

[28] Schmid SL, Ramachandran R, Surka M, Chappie JS, Fowler DM (2007). The dynamin middle domain is critical for tetramerization and higher-order self-assembly.. Embo J.

[29] Zhang PJ, Hinshaw JE (2001). Three-dimensional reconstruction of dynamin in the constricted state.. Nature Cell Biol.

[30] Hinshaw JE, Mears JA, Ray P (2007). A corkscrew model for dynamin constriction.. Structure.

[31] Chugh J, Sharma S, Hosur RV (2007). Pockets of short-range transient order and restricted topological heterogeneity in the guanidine-denatured state ensemble of GED of dynamin.. Biochemistry.

[32] Keller R (2004).

[33] Panchal SC, Bhavesh NS, Hosur RV (2001). Improved 3D triple resonance experiments, HNN and HN(C)N, for H-N and N-15 sequential correlations in (C-13, N-15) labeled proteins: Application to unfolded proteins.. J Biomol NMR.

[34] Farrow NA, Muhandiram R, Singer AU, Pascal SM, Kay CM (1994). Backbone dynamics of a free and phosphopeptide-complexed Src homology 2 domain studied by 15N NMR relaxation.. Biochemistry.

