# Supplementary figures and images for: NMR Derived Model of GTPase Effector Domain (GED) Self Association: Relevance to Dynamin Assembly
Source: PLoS One. 2012 Jan 12;7(1):e30109. doi: 10.1371/journal.pone.0030109 (PMC3257262; doi:10.1371/journal.pone.0030109)

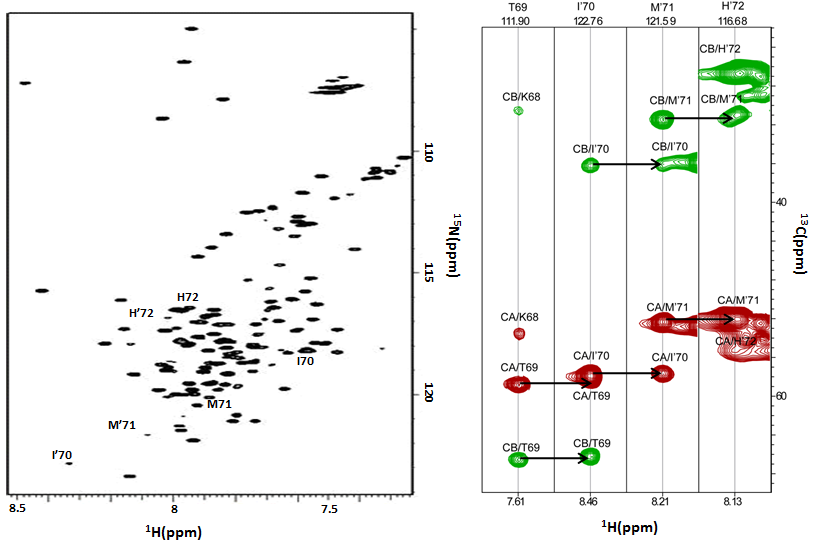

Supplement: Figure S1 — The stretch of residues (I70-H72) exhibiting two sets of peaks due to slow conformational exchange are marked on the HSQC spectrum of GED in 90% DMSO at 45°C as primed and unprimed residues. The sequential connectivities of the contiguous stretch of alternate residues (I′70-H′72) connected to T69 in the CBCANH experiment are shown. The red peaks indicate positive contour whereas green indicate negative contours. The arrows are connected between the centers of the peaks to indicate the connectivities between Cα and Cβ of i and i+1th residue. (TIF) [file pone.0030109.s001.tif]

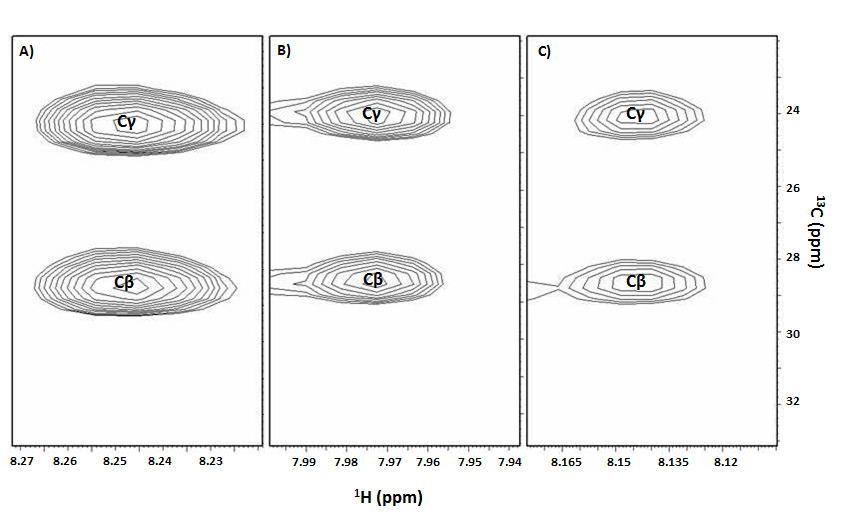

Supplement: Figure S2 — Illustrative strips from (H)C(CH)CONH TOCSY spectra of GED in 100% DMSO-d6 showing the 13C chemical shifts of (A) Pro 12′ (B) Pro 12 and (C) Pro 67. The chemical shift differences between the 13Cβ and 13Cγ nuclei are ∼5 ppm indicating trans conformation of the peptide bonds in the heterogeneous ensemble. (TIF) [file pone.0030109.s002.tif]

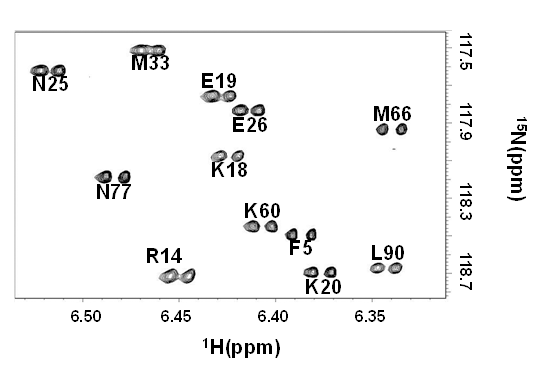

Supplement: Figure S3 — Selected region of the high resolution 1H-15N HSQC of GED at 90% DMSO to show the quality of the spectral resolution. Splitting in the peaks was used to measure the 3JHN-Hα coupling constants. (TIF) [file pone.0030109.s003.tif]
